# Interventions supporting medical practitioners in the provision of lactation care: A systematic review and narrative analysis

**DOI:** 10.1111/mcn.13160

**Published:** 2021-02-16

**Authors:** Melinda Boss, Nicole Saxby, Douglas Pritchard, Rafael Pérez‐Escamilla, Rhonda Clifford

**Affiliations:** ^1^ School of Allied Health, Division of Pharmacy The University of Western Australia Crawley Western Australia Australia; ^2^ Women's and Children's Services Royal Hobart Hospital Hobart Tasmania Australia; ^3^ School of Medicine, Division of General Practice The University of Western Australia Crawley Western Australia Australia; ^4^ Department of Social and Behavioral Sciences Yale School of Public Health New Haven Connecticut USA

**Keywords:** breastfeeding, lactation, medical practitioners, health intervention

## Abstract

Most children globally are not breastfed to recommendations. Medical practitioners are frequently visited in the first 6 months post‐partum, and the interaction at such visits significantly influences subsequent infant feeding decisions. Medical practitioners report that clinical practice in lactation is often disproportionately reliant on personal experience. This systematic review synthesises the literature on lactation health interventions used to support clinical decision making by medical practitioners. MEDLINE, Embase, PsycINFO, Scopus and Cochrane Library databases were searched for peer‐reviewed empirical studies published after 2000. Two reviewers independently screened and then assessed full‐text articles against inclusion criteria. Quality of reporting and risk of bias were independently assessed using three validated tools. No conclusions can be made regarding the success or failure of implementation strategies used or the outcomes of putting them into effect due to problems with study methodology, intervention reporting and risk of bias. Good‐quality research, which follows proven implementation frameworks, is needed to guide and sustain the incorporation of evidence‐based decision support into medical practitioners' care of breastfeeding mothers and infants.

**Key messages**

- Breastfeeding is not meeting recommendations globally with inconsistent advice contributing to early weaning.
- Medical practitioners influence infant feeding decisions but report reliance on personal experience for clinical decision making.
- Health interventions deliver evidence‐based decision support and improve consistency of care, but few lactation interventions are used by medical practitioners.
- No conclusions can be made regarding implementation strategies and outcomes of those reported due to poor study quality and bias.
- Good‐quality research is needed to guide and sustain the incorporation of evidence‐based decision support into medical practitioners' care of breastfeeding mothers and infants.

## 1 INTRODUCTION

Human lactation is well established as a foundation for human health (COAG Health Council, 2019). Breastfeeding contributes significantly to important health outcomes for both mother and infant that extend beyond the period of lactation and across the life course (Victora et al., 2016). The vast majority of children globally are not breastfed to recommendations, reducing survival, health and human capital outcomes (Walters et al., 2019). Inconsistent advice from health professionals is commonly reported by mothers to contribute to early weaning (Brodribb, 2012; Hauck et al., 2011; Pérez‐Escamilla, 2020; Simmons, 2002). Applying the evidence gained from research is a recognised method for promoting consistency of treatment and optimal outcomes (Institute of Medicine, 2000; Lodewijckx et al., 2012). Mothers and babies frequently visit medical practitioners in the first 6 months post‐partum (Gunn et al., 1996). The interaction at such visits significantly influences subsequent infant feeding decisions (Lu et al., 2001; Taveras et al., 2004). It is therefore of concern that doctors and medical specialists caring for breastfeeding women and infants report that they have not received the evidence‐based lactation information for the knowledge and skills expected of them (Brodribb et al., 2009; Moukarzel et al., 2020). Many report personal experience or the experiences of family and friends as a primary and most useful source of information (Brodribb et al., 2008; Finneran & Murphy, 2004; Gonzalez et al., 2014; Moukarzel et al., 2018; Pound et al., 2014). This indicates that clinical practice in lactation is often disproportionately reliant on experience and personal social networks.

Evidence‐based practice is defined as the integration of the best available research together with clinical expertise and patient values (Dawes et al., 2005). It follows that this requires knowledge of the evidence base by the medical practitioner. The gap between publication of new knowledge and its integration with practice is frequently acknowledged in health research, with many quoting the statement that the translation of new knowledge to practice takes approximately 17 years (Balas & Boren, 2000). Evidence‐based health interventions evolved from the evidence‐based medicine movement and aim to support clinical decision making by delivering the most appropriate research outcomes for particular clinical settings (Hailemariam et al., 2019). These health interventions are designed to improve professional practice and delivery of effective health services and include strategies designed to bring about changes in the behaviour of healthcare professionals (Effective Practice and Organisation of Care [EPOC], 2015).

The development of a health intervention does not necessarily translate into uptake by the targeted end user. A challenge for the health community is how to achieve this uptake (Peters et al., 2013). Implementation research aims to understand how to deliver these interventions effectively in practice and the different ways in which they are affected when they interact with the real world (Peters et al., 2013). This can be used to understand intervention processes and evaluate whether implementation is actually working (Peters et al., 2013). Understanding the practical challenges and lessons learnt from other initiatives helps to enhance efficiency when planning new health interventions (Proctor et al., 2011).

The results from this systematic review will be used to inform implementation strategies for LactaMap, an evidence‐based online lactation care support system designed to support consistent lactation care (Boss & Hartmann, 2019).

This systematic review aims to synthesise the literature on health interventions designed to support clinical decision making for medical practitioners in the specific clinical circumstance of lactation. The objectives are to

- identify lactation health interventions providing clinical decision support to individual medical practitioners,
- describe the implementation strategy and
- describe the implementation outcome variables reported.

## 2 METHODS

### 2.1 Protocol and registration

The systematic literature review was conducted in accordance with Preferred Reporting Items for Systematic Reviews and Meta‐Analyses (PRISMA) methodology with checklist available as an additional file (Appendix A) (Moher et al., 2009). The protocol for this review was registered with the International Prospective Register of Systematic Reviews (PROSPERO) (Review Number CRD42017070490).

### 2.2 Search strategy and study selection

A search strategy was devised in consultation with a health and medical sciences librarian from J Robin Warren Library at The University of Western Australia. Search terms and Medical Subject Headings (MeSH) chosen were those relevant to ‘doctor’ AND ‘lactation’ AND ‘intervention’. Databases searched were MEDLINE, Embase, PsycINFO, Scopus and Cochrane Library (Appendix B). The search was conducted on November 16, 2017, and rerun on February 6, 2019, and April 15, 2020.

### 2.3 Inclusion criteria

Publications were selected on the basis of the PICOS (Participants, Intervention, Comparator, Outcomes, Study design) outlined below (Huang et al., 2006). Implementation science was developed to facilitate the spread of evidence‐based practice (Bauer et al., 2015). The Sicily statement defining evidence‐based practice was published in 2005 (Dawes et al., 2005). It was therefore decided that studies published prior to 2000 would be unlikely to include newer concepts relating to interventions delivering research to practice. Accordingly, study inclusion was limited to those published from 2000 onwards.

#### 2.3.1 Participants

Studies were included if the primary intended user of the intervention was a generalist medical practitioner or specialist medical practitioner in the obstetric, gynaecological or paediatric specialty groupings as defined by World Health Organization (WHO) classification of health workers (WHO, 2020). Generalist medical practitioners include family and primary care doctors as well as general practice and family medicine doctors from countries where these are medical specialisations. This definition also included resident medical doctors training in these generalist or specialty groupings. Health professionals are defined broadly as individuals with knowledge and skills obtained from study at a higher education institution leading to the award of a first degree or higher qualification (WHO, 2020). Studies that reported on use of the intervention by other health professionals were included as long as the intervention had been designed with the medical practitioner as the primary targeted user and included data on their use. The context in which an intervention is delivered is an important consideration when describing implementation strategies and outcomes (Nilsen, 2015). Although behaviour‐change frameworks acknowledge the interaction between the individual and the organisational setting in which they work, theories used to analyse behaviour change of the individual are different from those applicable to a collective organisation (Nilsen, 2015). As uptake and adoption characteristics of the individual medical practitioner were of interest, interventions needed to be targeted to medical practitioners as an individual, rather than as a member of a collective (e.g., hospital policies that targeted all staff as a collective, of which medical practitioners were members, were excluded).

#### 2.3.2 Intervention

Based on the WHO International Classification of Health Interventions (WHO, 2015), the interventions of interest in this review were those designed to assess, improve, maintain or modify the functioning of human lactation by supporting clinical decision making. Human lactation is defined as a period of sustained milk synthesis, which requires frequent and effective removal of milk by the infant to function normally without any medical intervention or support (Boss & Hartmann, 2018). This means that the patient population expected to benefit from the intervention included the dyad of both mothers and infants.

#### 2.3.3 Comparator

The comparator was ‘usual practice’. Usual practice was indicated by no health intervention targeted to the medical practitioner to change current practice with respect to lactation.

#### 2.3.4 Outcome

Understanding implementation processes is assisted by conceptualising implementation outcomes (Proctor et al., 2011). Implementation outcomes help to indicate implementation success and implementation process. These outcomes indicate how well the target user engages with the intervention. A good health intervention will not produce successful clinical outcomes without target user engagement (Proctor et al., 2011). The distinction between implementation outcomes and clinical outcomes is important. Understanding implementation outcomes assists in determining whether failure of a health intervention is due to an ineffective health intervention or whether an efficacious intervention was deployed incorrectly (Proctor et al., 2011). Clinical outcomes relate specifically to treatment effectiveness and quality of care (Proctor et al., 2011). The outcomes of interest for this review were implementation outcomes (Peters et al., 2013; Proctor et al., 2011). Definitions first developed by Proctor et al. (2011), and modified by Peters et al. (2013), provided the taxonomy for describing the implementation outcome variables (Table 1).

**TABLE 1:** Implementation outcome variables with definitions (Peters et al., 2013; Proctor et al., 2011)

Implementation outcome variable	Definition
Acceptability	Perception amongst stakeholders that intervention is agreeable
Adoption	Intention to try to employ the intervention
Appropriateness	Perceived fit or relevance of the intervention for target audience
Feasibility	Extent to which the intervention can be carried out in the particular setting
Fidelity	Degree to which the intervention was implemented as designed
Implementation cost	Cost of delivery of the intervention
Coverage	Degree to which population eligible to benefit from the intervention actually receives it
Sustainability	Extent to which the intervention is maintained in a given setting

#### 2.3.5 Study criteria

Studies were limited to peer‐reviewed empirical research, defined as primary research based on experiment, observation or simulation (Hong, Gonzalez‐Reyes, & Pluye, 2018). This included studies that used quantitative, qualitative and mixed‐methods designs.

### 2.4 Exclusion criteria

Nonempirical articles including reviews and theoretical studies that gather data through critical studies, systematic review and meta‐analysis were excluded. Publications that only described a health intervention without any data on implementation outcomes were also outside the scope of this review.

Articles that were not available in English were removed, primarily due to resource limitations. Additionally, there is some evidence that restriction of language to English does not introduce systematic bias in systematic reviews of conventional medical fields (Morrison et al., 2012).

### 2.5 Data abstraction

Screening and data extraction were conducted using DistillerSR (Evidence Partners, Ottawa, Canada) web‐based systematic review software.

Data were independently abstracted by two reviewers (M. B. and N. S.) using a series of forms applied through DistillerSR. These forms were used to extract data regarding study design, implementation strategy, implementation outcome variables, quality of study methodology, quality of health intervention reporting and risk of bias. Forms were each piloted independently using three articles from the included references with disagreements discussed and resolved by consensus. If consensus could not be achieved, a third researcher (R. C.) was available for mediation. The two reviewers then independently extracted data from the remaining included articles for each form. Disagreements were resolved by discussion and moderation.

The form used for categorising study design was based on the Cochrane EPOC classification of study designs for evaluating the effect of healthcare interventions (EPOC, 2016).

Implementation science taxonomies are helpful to articulate both the strategies used to deliver a health intervention as well as the results of putting a health intervention into effect (Peters et al., 2013). In the context of implementation science, methods or techniques designed to deliver a health intervention are described variously as implementation interventions or implementation strategies (Curran et al., 2012; EPOC, 2015). In order to avoid confusion between a health intervention (which is the intervention designed to support lactation function) and an implementation intervention (the strategies employed to enhance uptake of the intervention designed to support lactation function by the medical practitioner), this literature review describes the strategies employed to enhance adoption as implementation strategies.

The form used to describe implementation strategies was based on the subset of strategies designed to bring about changes in the behaviour of individual healthcare professionals described by the EPOC taxonomy (EPOC, 2015). These included audit and feedback, clinical incident reporting, monitoring the performance of the delivery of healthcare, communities of practice, continuous quality improvement, educational games, educational materials, educational meetings, educational outreach visits, clinical practice guidelines, interprofessional education, local consensus processes, local opinion leaders, managerial supervision, patient‐mediated interventions, public release of performance data, reminders, routine patient‐reported outcome measures and tailored interventions (EPOC, 2015). Multiple implementation strategies indicate several interacting components to an intervention, increasing its complexity (Craig et al., 2013; Hawe, 2015).

### 2.6 Quality assessment

The quality of included studies was assessed using three different validated tools, one for quality of study methodology, one for quality of intervention reporting and one for risk of bias.

The methodological quality of the included studies was assessed using the Mixed Methods Appraisal Tool (MMAT) (Hong, Fàbregues, et al., 2018). MMAT permits appraisal of five categories of empirical studies: qualitative, randomised controlled trials, non‐randomised studies, quantitative descriptive and mixed‐methods studies. The first two screening questions in MMAT relate to whether there is a clear research question and whether collected data allow the research question to be addressed. These were used as additional criteria for exclusion of nonempirical studies. Studies that met all other inclusion criteria but did not meet these were excluded.

In addition to the quality of study methodology, good reporting of the health intervention is also required (Hoffmann et al., 2014). Description of the health intervention in sufficient detail allows replication, evidence synthesis and wider evaluation (Craig et al., 2013). The template for intervention description and replication (TIDieR) was used to appraise quality of reporting of the lactation health intervention (Hoffmann et al., 2014).

Risk of bias was assessed using the risk of bias in non‐randomised studies of interventions (ROBINS‐I) tool (Sterne et al., 2016). Studies that do not use randomisation to allocate interventions are often the main source of evidence regarding their impact. This is due to the difficulty in conducting randomised trials that are all embracing of a particular community (Sterne et al., 2016). ROBINS‐I allows evaluation of bias across seven domains, together with an overall judgement of bias for study outcome(s).

### 2.7 Data analysis

Due to the heterogeneity of included study methodologies as well as the complexity of the lactation interventions reported, a narrative synthesis of the data was used to describe the current state of knowledge.

## 3 RESULTS

### 3.1 Study selection

**FIGURE 1:**
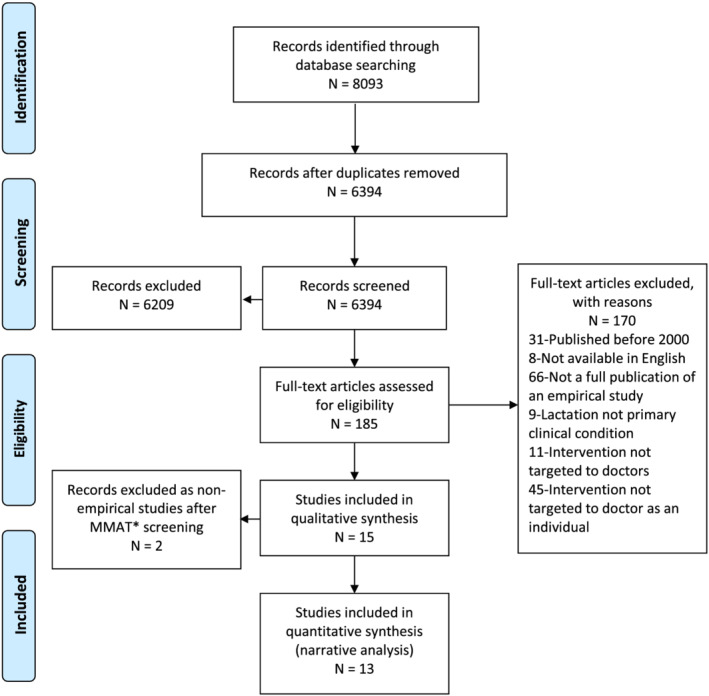
Preferred Reporting Items for Systematic Reviews and Meta‐Analyses (PRISMA) flow chart of qualitative synthesis used for study inclusion. *Mixed Methods Appraisal Tool

The search strategy identified 8093 records from database searches. After removal of duplicates, 6394 articles remained. Title, abstract and full‐text screening resulted in identification of 15 studies that met the qualitative synthesis inclusion criteria. The first two screening questions included in the MMAT resulted in exclusion of a further two studies for failing to meet the definition of an empirical study. A final total of 13 studies met the eligibility criteria for narrative analysis (Figure 1). Reference lists were hand searched but resulted in identification of no further articles.

The characteristics of studies reporting lactation interventions targeted to medical practitioners are summarised in Table 2.

**TABLE 2:** Summary of included studies

Author, year	Country	Sample size	Primary target for intervention	Control	Study design	Implementation strategy	Implementation outcome variable(s) reported
Albert et al., 2017	USA	45	Paediatric residents (first year)	No	Before–after study	Educational materials, educational meetings, interprofessional education, managerial supervision, tailored interventions	Acceptability, appropriateness
Burt et al., 2006	England	164	General practitioners	No	Mixed‐methods	Educational materials, educational outreach visits or academic detailing, interprofessional education, tailored interventions	Acceptability, adoption, appropriateness, feasibility
Feldman‐Winter et al., 2010	USA	417	Primary care residents	Yes	Non‐randomised trial	Educational materials, educational meetings, educational outreach visits or academic detailing, managerial supervision, patient‐mediated interventions	Acceptability, appropriateness, feasibility
Hillenbrand & Larsen, 2002	USA	49	Paediatrics and medicine/paediatrics residents (first–fourth years)	No	Before–after study	Educational materials, educational meetings, interprofessional education, patient‐mediated interventions	Acceptability, adoption, appropriateness, feasibility, fidelity, coverage
Holmes et al., 2012	USA	39	Residents and faculty	Yes	Non‐randomised trial	Audit and feedback, educational meetings, interprofessional education, managerial supervision	Acceptability, adoption, appropriateness, feasibility, fidelity
O'Connor et al., 2011	USA	3456	Residents, physicians and mid‐level providers	No	Before–after study	Educational materials, interprofessional education	Appropriateness
Ogburn et al., 2005	USA	24	Medical students and paediatric, obstetrics and gynaecology and family medicine residents	No	Noncomparative study	Monitoring of the performance of the delivery of healthcare, educational materials, educational meetings, interprofessional education	Acceptability, appropriateness, feasibility, fidelity, implementation cost
Shen & Rudesill, 2016	USA	43	Paediatric residents	No	Before–after study	Educational meetings	Adoption, appropriateness, fidelity, coverage
Tender et al., 2014	USA	39	Paediatric residents (first year)	No	Before–after study	Educational materials, interprofessional education, patient‐mediated interventions	Acceptability, adoption, appropriateness, feasibility
Velillas et al., 2007	Spain	42	Paediatric residents	No	Before–after study	Educational materials, patient‐mediated interventions	Acceptability, adoption, appropriateness, feasibility
Bunik et al., 2006	USA	40	Resident doctors	Yes	Non‐randomised trial	Educational materials, interprofessional education, patient‐mediated interventions	Acceptability, adoption, appropriateness, feasibility, implementation cost
Ingram, 2006	England	50	General practitioners	No	Before–after study	Educational materials, educational meetings, tailored interventions	Acceptability, adoption, appropriateness, feasibility, implementation cost
Srinivasan et al., 2014	Canada	162	Family medicine residents and practising family physicians	No	Before–after study	Educational meetings	Acceptability, appropriateness

### 3.2 Lactation intervention description/characteristics

The rationale for all lactation interventions reported in the included studies was an identified inadequacy in breastfeeding knowledge, training or education in the participant population. Albert et al. (2017) also identified that breastfeeding education had previously proven feasible in the participant population. The elements reported to be essential to the interventions were varied. Two studies (Albert et al., 2017; Shen & Rudesill, 2016) reported none beyond the need to deliver breastfeeding education. Essential elements reported in other studies included time efficiency (Srinivasan et al., 2014; Tender et al., 2014; Velillas et al., 2007), online or e‐learning (O'Connor et al., 2011; Velillas et al., 2007), practice‐based learning (Ingram, 2006), interactive training techniques (Hillenbrand & Larsen, 2002), need to fit existing curriculum structure (Holmes et al., 2012), team‐teaching approach (Burt et al., 2006), use of a field trip design model (Bunik et al., 2006; Feldman‐Winter et al., 2010) and the aim to achieve a stated level of breastfeeding awareness (Ogburn et al., 2005).

### 3.3 Implementation strategies

Implementation strategies employed by each study are described in Table 2. All but two studies (Shen & Rudesill, 2016; Srinivasan et al., 2014) employed multiple implementation strategies. The EPOC taxonomy for implementation strategies targeted to healthcare workers identifies 19 possible strategies. Two studies (Albert et al., 2017; Feldman‐Winter et al., 2010) incorporated five strategies, four studies incorporated four strategies (Burt et al., 2006; Hillenbrand & Larsen, 2002; Holmes et al., 2012; Ogburn et al., 2005), three studies used three strategies (Bunik et al., 2006; Ingram, 2006; Tender et al., 2014) and four studies used two or fewer implementation strategies (O'Connor et al., 2011; Shen & Rudesill, 2016; Srinivasan et al., 2014; Velillas et al., 2007). Educational materials that contained knowledge to support care were the most common strategy used, employed in 10 of the 13 studies (Albert et al., 2017; Bunik et al., 2006; Burt et al., 2006; Feldman‐Winter et al., 2010; Hillenbrand & Larsen, 2002; Ingram, 2006; O'Connor et al., 2011; Ogburn et al., 2005; Tender et al., 2014; Velillas et al., 2007). Educational meetings and interprofessional education involving more than one health profession were the next most frequently used strategies, each used in eight of the included studies (Table 2).

Multiple implementation strategies were utilised in 11 of the 13 studies (Albert et al., 2017; Bunik et al., 2006; Burt et al., 2006; Feldman‐Winter et al., 2010; Hillenbrand & Larsen, 2002; Holmes et al., 2012; Ingram, 2006; O'Connor et al., 2011; Ogburn et al., 2005; Tender et al., 2014; Velillas et al., 2007). This indicates that the lactation interventions reported were typically complex, having several interacting components all acting in the system or context in which they were placed (Hawe, 2015).

### 3.4 Implementation outcome variables

Implementation outcome variables reported are also listed in Table 2. All included studies reported on the medical practitioner's perceived appropriateness of the lactation intervention for its fit, relevance or compatibility in the given practice setting. All but two of the studies (O'Connor et al., 2011; Shen & Rudesill, 2016) reported on acceptability, which related to whether medical practitioners perceived the intervention as agreeable. Nine studies reported on feasibility, which considered the actual fit or suitability of the intervention in the particular health setting (Bunik et al., 2006; Burt et al., 2006; Feldman‐Winter et al., 2010; Hillenbrand & Larsen, 2002; Holmes et al., 2012; Ingram, 2006; Ogburn et al., 2005; Tender et al., 2014; Velillas et al., 2007). Eight studies reported on adoption, which related to the initial intention or decision to try the intervention (Bunik et al., 2006; Burt et al., 2006; Hillenbrand & Larsen, 2002; Holmes et al., 2012; Ingram, 2006; Shen & Rudesill, 2016; Tender et al., 2014; Velillas et al., 2007). Of eight possible implementation outcome variables, nine studies reported on four or more variables (Bunik et al., 2006; Burt et al., 2006; Hillenbrand & Larsen, 2002; Holmes et al., 2012; Ingram, 2006; Ogburn et al., 2005; Shen & Rudesill, 2016; Tender et al., 2014; Velillas et al., 2007). Three studies reported on two or fewer implementation outcome variables (Burt et al., 2006; O'Connor et al., 2011; Srinivasan et al., 2014).

Most of the lactation interventions in this review were novel, delivering new educational content or incorporating previously developed content in a new way (Albert et al., 2017; Bunik et al., 2006; Burt et al., 2006; Feldman‐Winter et al., 2010; Hillenbrand & Larsen, 2002; Holmes et al., 2012; Ingram, 2006; Ogburn et al., 2005; Shen & Rudesill, 2016; Tender et al., 2014; Velillas et al., 2007). The outcomes reported in these studies reflected this, with few reporting on fidelity, implementation cost or coverage (Bunik et al., 2006; Hillenbrand & Larsen, 2002; Holmes et al., 2012; Ingram, 2006; Ogburn et al., 2005; Shen & Rudesill, 2016) and none reporting on sustainability—which can require some follow‐up of an established intervention over time.

The findings from these studies show that the number of implementation strategies reported ranged from one to five and the number of implementation outcome variables reported ranged from one to six, but it was not possible to find a consistent pattern of results.

### 3.5 Quality of reporting

Quality of study methodology and quality of reporting of the intervention description were assessed.

For appraisal of quality of study methodology, 12 study designs defined as either before and after studies, non‐randomised trials and noncomparative studies using EPOC criteria were all classified as quantitative non‐randomised studies according to criteria defined in the MMAT (Hong, Fàbregues, et al., 2018). The developers of MMAT recommend against presentation of a single overall quality score alone but acknowledge that this can be useful when used as an addition to a descriptive summary of MMAT criteria (Hong, 2020). An overall score calculated as a percentage of quality criteria met was included based on these recommendations to assist in reporting of these results. Study methodology was generally poor. Of the included studies, three met 60% of the quality criteria, six met 20–40% and four met none (Table 3).

**TABLE 3:** Results of methodological quality assessment using the Mixed Methods Appraisal Tool (Hong, Fàbregues, et al., 2018)

	Quantitative non‐randomised study					
	1. Are participants representative of the target population?	2. Are measurements regarding both the outcome and the intervention appropriate?	3. Are there complete outcome data?	4. Are confounders accounted for in design and analysis?	5. Was the intervention administered as intended during the study period?	% quality criteria met
Albert et al., 2017	Yes	Yes	Yes	No	Can't tell	60
Feldman‐Winter et al., 2010	Yes	Yes	Yes	No	Can't tell	60
Hillenbrand & Larsen, 2002	Can't tell	Can't tell	No	No	No	0
Holmes et al., 2012	Yes	Can't tell	No	Can't tell	No	20
O'Connor et al., 2011	Can't tell	Can't tell	No	No	No	0
Ogburn et al., 2005	Yes	No	No	No	No	20
Shen & Rudesill, 2016	No	No	No	No	No	0
Tender et al., 2014	Yes	Can't tell	Yes	No	Yes	60
Velillas et al., 2007	Can't tell	No	Can't tell	No	Yes	20
Bunik et al., 2006	Can't tell	No	Yes	No	Yes	40
Ingram, 2006	Can't tell	No	No	No	Can't tell	0
Srinivasan et al., 2014	Can't tell	Can't tell	No	No	Yes	20
	**Mixed‐methods study**					
	1. Is there adequate rationale for using a mixed‐methods design?	2. Are the different components effectively integrated?	3. Are the outputs of integrated qualitative and quantitative components adequately interpreted?	4. Are divergencies and inconsistencies between quantitative and qualitative results adequately addressed?	5. Do the different study components adhere to the quality criteria of each tradition of the methods involved?	% quality criteria met
Burt et al., 2006	Yes	Yes	Can't tell	Can't tell	No	40

The quality of reporting of the health intervention implemented was variable when assessed according to TIDieR criteria (Table 4). All studies provided a name or brief description of the intervention as well as some rationale, theory or goal of elements essential to the intervention. All studies reported that the rationale for intervention development was an identified need for lactation education. No studies reported all recommended criteria.

**TABLE 4:** Quality of reporting of health interventions according to template for intervention description and replication criteria (Hoffmann et al., 2014)

	Albert et al., 2017	Burt et al., 2006	Feldman‐Winter et al., 2010	Hillenbrand & Larsen, 2002	Holmes et al., 2012	O'Connor et al., 2011	Ogburn et al., 2005	Shen & Rudesill, 2016	Tender et al., 2014	Velillas et al., 2007	Bunik et al., 2006	Ingram, 2006	Srinivasan et al., 2014
**Brief name**													
Provide a name or phrase that describes the intervention	Y	Y	Y	Y	Y	Y	Y	Y	Y	Y	Y	Y	Y
**Why**													
Describe rationale, theory or goal of elements essential to the intervention	Y	Y	Y	Y	Y	Y	Y	Y	Y	Y	Y	Y	Y
**What (materials)**													
Describe physical or informational materials used in the intervention	Y	Y	Y	Y	Y	Y	?	Y	Y	Y	Y	Y	N
**What (procedures)**													
Describe procedures, activities and/or processes used in the intervention	Y	Y	Y	Y	Y	Y	?	?	Y	Y	Y	Y	N
**Who provided**													
Describe expertise, background and any specific training given for intervention provider(s)	Y	?	Y	?	?	?	Y	?	?	?	?	?	N
**How**													
Modes of intervention delivery and whether provided individually or in a group	Y	Y	Y	Y	Y	Y	Y	Y	?	?	?	Y	N
**Where**													
Type(s) of location(s) where intervention occurred including necessary infrastructure or relevant features	?	Y	Y	?	Y	?	Y	?	Y	Y	?	Y	?
**When and how much**													
Describe number of times intervention delivered and over what period of time including number of sessions, their schedule, duration and intensity	Y	?	Y	?	Y	Y	?	?	Y	Y	?	Y	?
**Tailoring**													
If intervention tailoring was planned, describe what, why, when and how	?	?	?	NA	NA	?	NA	NA	NA	NA	NA	?	NA
**Modification**													
If intervention was modified, describe the changes	?	?	?	?	?	?	?	?	?	?	?	?	N
**How well (planned)**													
If intervention adherence or fidelity was assessed, describe how and by whom, and if strategies were used to maintain or improve fidelity, describe them	?	?	?	?	?	?	?	?	?	?	?	?	N
**How well (actual)**													
If intervention adherence or fidelity was assessed, describe extent to which it was delivered as planned	?	?	?	?	?	?	NA	?	?	?	?	?	N

*Note*: ‘?’ indicates that the item was not reported/not sufficiently reported; ‘N/A’ indicates that the item was not applicable to the intervention reported; and ‘Y’ indicates that the item was reported.

### 3.6 Risk of bias

**FIGURE 2:**
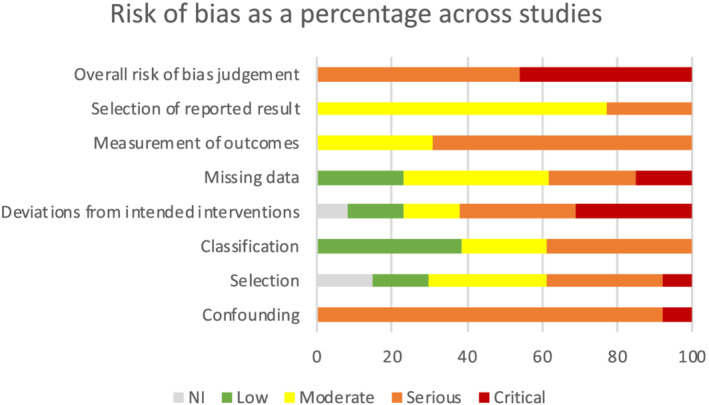
Risk of bias in non‐randomised studies of interventions as a percentage across studies (*NI* indicates not enough information to make a judgement; *low* indicates comparable with a well‐performed randomised trial; *moderate* indicates sound for a non‐randomised study, but not comparable with a well‐performed randomised trial; *serious* indicates presence of important problems; *critical* indicates too problematic to provide useful evidence on the effects of the intervention; and *overall risk of bias* is equal to the most severe level of bias found in any domain)

Overall risk of bias was serious (indicating presence of important problems) or critical (too problematic to provide useful evidence of intervention effect) within all included studies (Figure 2). Confounding bias was the primary source. Bias in measurement of outcomes and selection of reported results were also significant contributors with all studies assessed as having moderate to serious bias for these two domains.

Thus, the outcomes of these studies are unlikely to be reliable.

## 4 DISCUSSION

The findings of this systematic review highlight a need for high‐quality implementation research on lactation interventions for medical practitioners caring for breastfeeding families. The limited studies reporting on lactation interventions all identified a need for medical practitioner lactation education, but poor quality of study methodology and reporting and serious to critical risk of bias precluded further conclusions from being made. As evidence‐based knowledge has been shown to improve consistency of medical care and optimise outcomes (Institute of Medicine, 2000; Lodewijckx et al., 2012), it is not surprising that when this education is lacking, mothers are reporting that inconsistent advice is contributing to early weaning (Brodribb, 2012; Hauck et al., 2011; Pérez‐Escamilla, 2020; Simmons, 2002).

### 4.1 Implementation strategies

Consistent with expectations, use of multiple implementation strategies indicated that the lactation interventions reported were typically complex (Medical Research Council, 2008; Pérez‐Escamilla & Hall Moran, 2016). Intervention complexity increases the challenges in designing good‐quality studies to understand outcomes (Hawe, 2015; Medical Research Council, 2008; O'Cathain et al., 2019; Paina & Peters, 2012). Guidance developed by the United Kingdom's Medical Research Council (MRC) for complex intervention development and evaluation provides framework to assist researchers in identifying and overcoming these challenges (Medical Research Council, 2008). For example, randomised controlled trials can work well for simple interventions such as testing the efficacy of a new drug where the intervention (new drug) is directly linked with the outcome (therapeutic effect). However, they can be problematic for complex interventions. Adaptation to a local setting may allow a complex intervention to work better than strict fidelity to a predefined protocol, as long as the function it performs remains the same (Hawe, 2015; Medical Research Council, 2008). Carefully planned research following proven frameworks to assist the design of non‐randomised intervention studies, such as those provided by the United Kingdom's MRC, is urgently needed if lactation interventions for medical practitioners are to be used.

### 4.2 Implementation outcomes

Understanding the implementation outcome variables involved in delivering health interventions helps to evaluate their efficacy (Fixsen et al., 2005; Peters et al., 2013). However, these outcomes do not necessarily have equal importance during the process of health intervention delivery. Much can be learned when an intervention is first designed. Studies reporting implementation of new interventions can serve as entry points to show how such interventions work in a particular context. Novel or new interventions typically focus on outcomes relating to acceptability, adoption, appropriateness and feasibility (Peters et al., 2013). This was reflected in the studies identified by this review. Only two studies (O'Connor et al., 2011; Srinivasan et al., 2014) reported on existing interventions that were not novel. Further, no studies reported on sustainability, meaning there was little evidence to inform continuity of intervention use over time.

### 4.3 Quality of study methodology and intervention reporting

Although inclusion of a range of study designs can be appropriate for review of complex interventions, good study methodology is critical to enable conclusions to be drawn from the outcomes (Hong, Gonzalez‐Reyes, et al., 2018). Quality of study methodology was generally poor, with only three (Albert et al., 2017; Feldman‐Winter et al., 2010; Tender et al., 2014) including greater than 50% of criteria required for good empirical studies. Use of appropriate quality appraisal tools to inform methodology during the design phase of intervention studies would help mitigate these shortcomings.

Previous research has found consistent inadequacies in the reporting of complex interventions to improve health (Datta & Petticrew, 2013). Inadequacies identified include the need to provide more detailed intervention descriptions, using theory in intervention design, ensuring fidelity and capturing multiple outcomes (Datta & Petticrew, 2013; Hawe, 2015). These were also reflected in this review, with nine studies meeting only 50% or less of the criteria required for quality intervention reporting (Bunik et al., 2006; Burt et al., 2006; Hillenbrand & Larsen, 2002; O'Connor et al., 2011; Ogburn et al., 2005; Shen & Rudesill, 2016; Srinivasan et al., 2014; Tender et al., 2014; Velillas et al., 2007). Complex interventions can be challenging to report adequately where descriptions may be restricted by word limits. Suggestions for addressing this include publication of the intervention development process as a manual made available online, or as an additional publication describing the process, which can be particularly useful if multiple lessons were learned during development (O'Cathain et al., 2019).

### 4.4 Risk of bias

In addition to poor study methodology and reporting of interventions, the ability to draw firm conclusions about implementation of lactation interventions targeted to medical practitioners was further limited by study bias. Non‐randomised studies of interventions are recognised as having the potential to deliver evidence critical for intervention evaluation, but bias impairs the ability to determine the likely impact of outcomes reported (Sterne et al., 2016). Six studies were assessed as having critical risk of bias, meaning that they were too problematic to provide useful evidence for intervention effect (Hillenbrand & Larsen, 2002; Holmes et al., 2012; O'Connor et al., 2011; Ogburn et al., 2005; Srinivasan et al., 2014; Velillas et al., 2007). The remaining seven studies had a serious overall risk of bias assessment, meaning that important problems limited the ability to extract useful evidence for review synthesis (Albert et al., 2017; Bunik et al., 2006; Burt et al., 2006; Feldman‐Winter et al., 2010; Ingram, 2006; Shen & Rudesill, 2016; Tender et al., 2014). Confounding bias was the largest contributor to study bias. As only three studies included a control group (Bunik et al., 2006; Feldman‐Winter et al., 2010; Holmes et al., 2012), inclusion of a control group is an obvious suggestion for reduction of this bias. Blinding of outcome assessors to intervention status would reduce the next largest contributor to bias, which was bias in measurement of outcomes.

### 4.5 Strengths and limitations

This review used a systematic approach, following the PRISMA checklist with PROSPERO protocol registration. The review focused specifically on implementation of lactation interventions targeted to the medical practitioner as an individual, rather than as a member of a collective. This meant that implementation strategies and outcomes could be described in terms of the stakeholder directly targeted (medical practitioners themselves as opposed to the organisations that employ them). Use of three validated tools to comprehensively assess the quality of included studies allowed detailed insight into the strength of evidence reported.

Conclusions were limited by the presence of serious to critical risk of bias in all studies, which was compounded by poor quality in study methodology and intervention reporting. Although this meant that there was little to inform specific implementation strategies for LactaMap, the value of using theory‐based models or frameworks to help inform study design and mitigate the challenges of complex intervention development and evaluation was highlighted (Nilsen, 2015).

## 5 CONCLUSION

This systematic review highlighted the deficit in medical practitioner lactation interventions and indicates an urgent need for high‐quality research on their uptake in practice. No conclusions can be made regarding the success or failure of implementation strategies used or the outcomes of putting them into effect due to problems with study methodology, intervention reporting and risk of bias.

This has several implications for further research. Breastfeeding mothers and infants are not meeting recommendations globally. Medical practitioners supporting them are reporting that clinical practice is overly reliant on personal experience and social networks, which is likely contributing to inconsistent care. Few lactation interventions have been implemented to support clinical decision making. Good‐quality research, which follows proven implementation frameworks, is needed to guide and sustain the incorporation of evidence‐based decision support into medical practitioners' care of breastfeeding mothers and infants (Pérez‐Escamilla & Odle, 2019).

## CONFLICTS OF INTEREST

The authors declare that they have no conflicts of interest.

## CONTRIBUTIONS

MB developed the search question and strategy with considerable input and supervision from RC, DP and RP‐E. MB conducted the search. MB and NS selected full‐text articles for eligibility, abstracted data and assessed quality of evidence of the selected articles. MB wrote the manuscript and developed figures and tables with review and input from RC, DP and RP‐E. All authors gave final approval of drafts and final version of this manuscript.

## Data Availability

The data that support the findings of this study are available from the corresponding author upon reasonable request.

## APPENDIX B

**TABLE B1:** Search terms

MEDLINE		
physician*.mp	Lactation/	intervention.mp
Physicians/	Lactation Disorders/	Early Medical Intervention/
Physicians, Family/	Milk, Human/	Evidence‐Based Medicine/
doctor*.mp	breast feed*.mp	Practice Guideline/
obstetric*.mp	Breast Feeding/	Clinical Protocols/
gyn?ecolog*.mp	breastfeed*.mp	Critical Pathways/
Gynecology/	‘human milk’.mp	Education, Medical/
Obstetrics/	lactation.mp	‘Delivery of Health Care’/
p?ediatric*.mp	breastfed.mp	protocol*.mp
Pediatrics/		guideline*.mp
Registrar*.mp		
General Practitioners/		
**Embase**		
physician*.mp	lactation/	intervention.mp
physician/	Lactation.mp	lactation disorder/
doctor*.mp	breastfeed*.mp	early intervention/
general practitioner/	breast feeding/	evidence based medicine/
general practitioner*.mp	‘breast feed*’.mp	practice guideline/
obstetric*.mp	breast milk/	guideline*.mp
obstetrics/	‘human milk’.mp	protocol*.mp
gynecology/	lactation.mp	clinical protocol/
gyn?ecolog*.mp	breastfed.mp	clinical pathway/
pediatrician/		medical education/
p?ediatric*.mp		health care delivery/
Registrar*.mp		
**PsycINFO**		
physician*.mp	exp LACTATION/	intervention*.mp
exp PHYSICIANS/	breastfeed*.mp	‘evidence based medicine’.mp
exp Family Physicians/	exp Breast Feeding/	exp Evidence Based Practice/
doctor*.mp	‘breast feed*’.mp	exp Treatment Guidelines/
obstetric*.mp	‘human milk’.mp	guideline*.mp
exp OBSTETRICS/	lactation.mp	protocol*.mp
gyn?ecolog*.mp	breastfed.mp	exp Health Promotion/
p?ediatric*.mp		exp Medical Education/
exp PEDIATRICIANS/		exp Health Care Delivery/
registrar.mp		
‘general practitioner*’.mp		
exp General Practitioners/		
**Cochrane Central Register of Controlled Trials**		
physician*.mp	lactation.mp	intervention*.mp
Physicians, Family/	Lactation/	Early Medical Intervention/
doctor*.mp	Lactation Disorders/	Evidence‐Based Medicine/
obstetric*.mp	breastfeed*.mp	‘evidence based medicine’.mp
Obstetrics/	Breast Feeding/	Practice Guidelines as Topic/
gyn?ecolog*.mp	‘breast feed*’.mp	guideline*.mp
Gynecology/	‘human milk’.mp	protocol*.mp
p?ediatric*.mp	Milk, Human/	Clinical Protocols/
Pediatrics/	breastfed.mp	Critical Pathways/
registrar*.mp		Education, Medical/
Family Practice/		‘Delivery of Health Care’/
‘general practitioner*’.mp		
**Cochrane Database of Systematic Reviews**		
physician*.mp	lactation.mp	intervention*.mp
doctor*.mp	breastfeed*.mp	guideline*.mp
obstetric*.mp	‘breast feed*’.mp	protocol*.mp
gyn?ecolog*.mp	‘human milk’.mp	‘clinical protocol’.mp
p?ediatric*.mp		‘critical pathway’.mp
registrar*.mp		‘medical education’.mp
‘general practitioner*’.mp		‘delivery of health care’.mp
**Scopus**		
physician*	lactation	intervention*
doctor*	breastfeed*	‘evidence based medicine’
obstetric*	‘breast feed*’	‘practice guideline*’
gyn?ecolog*	‘human milk’	‘clinical protocol*’
p?ediatric*	breastfed	education
Registrar*		
‘general practitioner*’		

*Note*: The search terms within each column were combined with ‘OR’ and between each column were combined with ‘AND’. ‘Dirty’ searches of the literature excluded relevant articles when ‘implementation’ and related synonyms were included as keywords. In order to capture those articles, ‘implementation’ was not included in the search terms.
